# Multi-*phase*ted problems of TDP-43 in selective neuronal vulnerability in ALS

**DOI:** 10.1007/s00018-021-03792-z

**Published:** 2021-03-11

**Authors:** Kazuhide Asakawa, Hiroshi Handa, Koichi Kawakami

**Affiliations:** 1 Department of Chemical Biology, Tokyo Medical University, Shinjuku-ku, Tokyo 160-8402 Japan; 2 Division of Molecular and Developmental Biology, National Institute of Genetics, 1111 Yata, Mishima, Shizuoka 411-8540 Japan; 3 Department of Genetics, Graduate University for Advanced Studies (SOKENDAI), 1111 Yata, Mishima, Shizuoka 411-8540 Japan

## Abstract

Transactive response DNA-binding protein 43 kDa (TDP-43) encoded by the *TARDBP* gene is an evolutionarily conserved heterogeneous nuclear ribonucleoprotein (hnRNP) that regulates multiple steps of RNA metabolism, and its cytoplasmic aggregation characterizes degenerating motor neurons in amyotrophic lateral sclerosis (ALS). In most ALS cases, cytoplasmic TDP-43 aggregation occurs in the absence of mutations in the coding sequence of *TARDBP*. Thus, a major challenge in ALS research is to understand the nature of pathological changes occurring in wild-type TDP-43 and to explore upstream events in intracellular and extracellular milieu that promote the pathological transition of TDP-43. Despite the inherent obstacles to analyzing TDP-43 dynamics in in vivo motor neurons due to their anatomical complexity and inaccessibility, recent studies using cellular and animal models have provided important mechanistic insights into potential links between TDP-43 and motor neuron vulnerability in ALS. This review is intended to provide an overview of the current literature on the function and regulation of TDP-43-containing RNP granules or membraneless organelles, as revealed by various models, and to discuss the potential mechanisms by which TDP-43 can cause selective vulnerability of motor neurons in ALS.

## Introduction

Abnormal aggregation of proteins that normally function as components of ribonucleoprotein (RNP) granules is a hallmark of neurodevelopmental and neurodegenerative diseases [1–3]. Under normal physiological conditions, RNP granules form membraneless partitions in the nucleus and cytoplasm to control the flow of genetic information. Assembly of RNP granules may also be driven by external stimuli, such as during stress granule (SG) formation in response to cellular stresses. A remarkable feature of RNP granules is their compositional heterogeneity and structural flexibility. A single RNP, depending on the interacting partner (proteins or RNAs), can form either liquid-like physiological assemblies or solid-like fibers [4, 5]. Such solid-like RNPs are a candidate source for pathological aggregates that accumulate over time during the progression of diseases, although some evidence suggests that protein aggregates without RNA are associated with cellular toxicity [6, 7].

Transactive response DNA-binding protein 43 kDa (TDP-43) is an evolutionarily conserved RNA/DNA-binding protein encoded by the *TARDBP* gene and regulating transcription [8–10], RNA metabolism [11–14], anti-viral response [15], DNA damage response [16], and chromatin structure [17]. In 97% of amyotrophic lateral sclerosis (ALS) cases, the most common motor neuron disease, and in 45% of frontotemporal dementia (FTD) cases [18], aggregation of TDP-43 is detectable in degenerating neurons. TDP-43 protein has a homo-oligomerization domain, RNA-binding domains, and an intrinsically disordered region (IDR) in tandem, each containing amino acid sequence motifs for the various regulation, such as post-translational modification, nucleocytoplasmic transport, and proteolysis. In sporadic ALS, which accounts for approximately 90% of ALS cases, TDP-43 aggregation occurs without mutation in the coding sequence of the *TARDBP* gene, rendering the mechanism underlying aggregation of wild-type TDP-43 largely unknown. On the other hand, in familial ALS cases associated with the *TARDBP* locus, mutations have mostly, but not exclusively, been found in the IDR [19, 20]. Given that by mediating a multitude of intermolecular interactions, IDRs typically drive the transition from soluble protein to liquid droplets of protein [21], dysregulation of IDR-dependent homomeric and heteromeric TDP-43 assembly likely underlies the pathogenesis of ALS. Under physiological conditions, TDP-43-containing RNP granules exist in various subcellular compartments, each granule differing in its protein: RNA composition according to its function. Currently, an understanding of how a cell monitors the global and local levels of intracellular TDP-43 and specifies division of labor for granular and non-granular TDP-43 is far from complete.

**Fig. 1:**
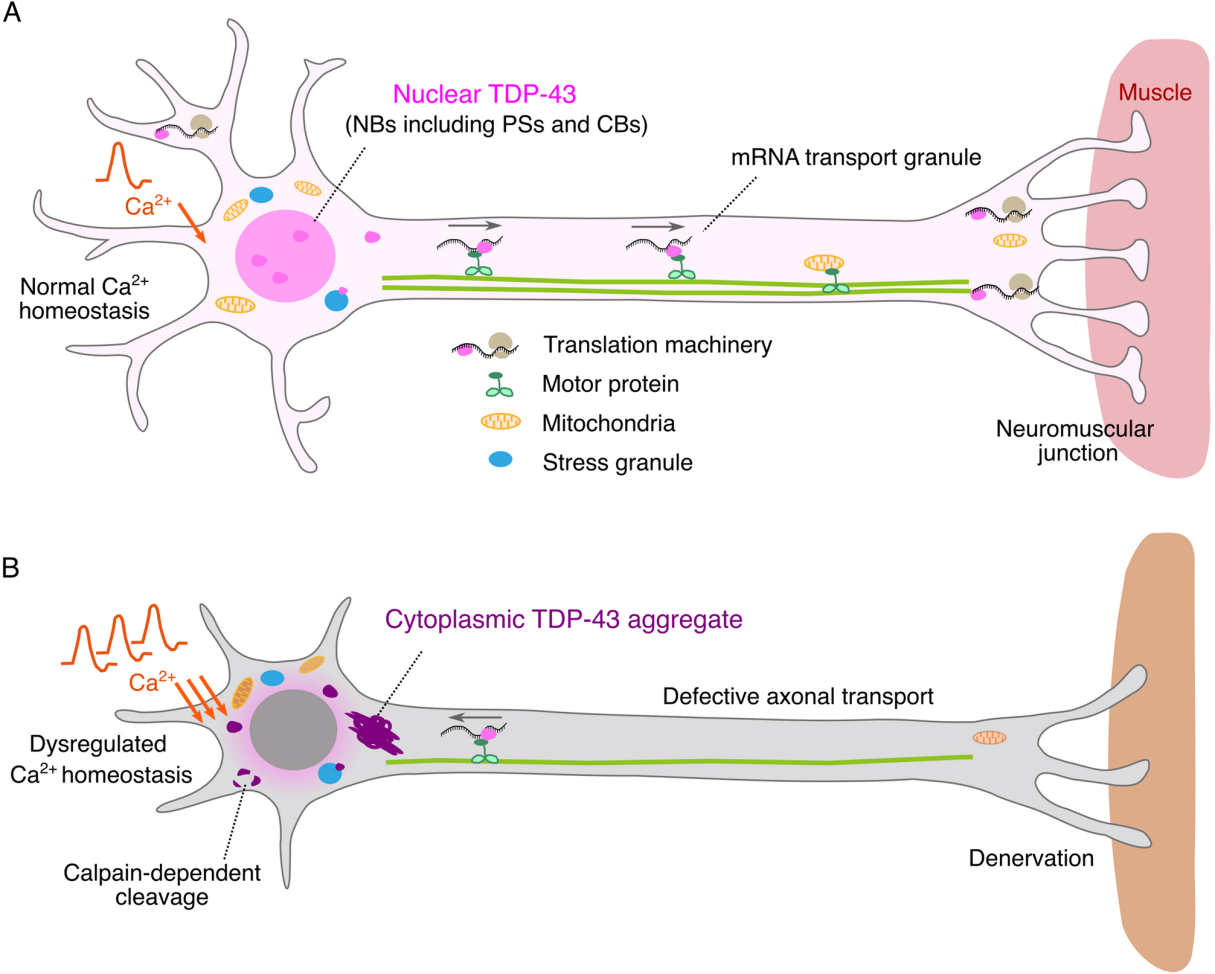
Diagrams depicting TDP-43 in motor neurons under physiological (**a**) and pathological (**b**) conditions. **a** Under physiological conditions, TDP-43 (magenta) is primarily nuclear and regulates transcription and pre-mRNA processing. TDP-43 also forms mRNA transport granules and supports translation at neuromuscular synapses. Intracellular Ca^2+^ homeostasis is maintained by mitochondria. **b** In sporadic ALS or familial ALS associated with *TARDBP* mutations, the cytoplasmic pool of TDP-43 increases and forms aggregates (purple). The nuclear pool of TDP-43 is instead depleted. Transport granules containing pathological TDP-43 frequently display retrograde movement, which may result in diminished translation in the synaptic terminal and denervation. Dysregulated Ca^2+^ homeostasis and/or excessive Ca^2+^ influx due to neuronal hyperactivation may promote calpain-dependent TDP-43 cleavage, promoting TDP-43 aggregation. Neuronal hyperactivation produces the aggregation-prone splice isoforms of TDP-43

At the systems level, TDP-43 is a ubiquitously expressed protein, like the misfolded proteins in other neurodegenerative diseases, such as amyloid β in Alzheimer’s disease, α-Synuclein in Parkinson’s disease, and huntingtin in Huntington’s disease [22]. However, as observed in these neurodegenerative diseases, selective subpopulations of neurons are affected in ALS: upper and lower motor neurons. Despite being a pathological hallmark of ALS, the extent to which TDP-43 aggregation accounts for the selective vulnerability of motor neurons is largely unknown. This is primarily due to the anatomical complexity and inaccessibility of motor neurons, hampering in vivo investigation of TDP-43 dynamics in live motor neurons [23, 24]. Thus, for an understanding of the pathogenesis of ALS associated with TDP-43 aggregation, it is imperative to fully figure out the functions and regulation of TDP-43-containing RNP complexes that assemble in the normal physiological conditions, investigate the nature of pathological changes occurring in wild-type TDP-43, and explore upstream intracellular and extracellular factors that promote the pathological transition of TDP-43 in motor neurons in vivo (Fig. 1). With these challenges in mind, in the present review, we present an overview of the domain structure of TDP-43 and its regulation. We subsequently discuss the known functions and properties of TDP-43 granules that have been elucidated via numerous cell culture and animal models. Finally, we review neuron-specific TDP-43 properties to explore the potential link between TDP-43 pathology and selective neuronal vulnerability in ALS.

## DNA and RNA-binding functions of TDP-43

TDP-43, a ubiquitous DNA/RNA binding protein, plays multiple roles in both the nucleus and cytoplasm. First identified as a cellular factor that bound to a regulatory element of the human immunodeficiency virus type 1 (HIV-1) long terminal repeat (LTR) and repressed its transcription [8], TDP-43 was later characterized as a binding protein for the spermatid-specific promoter of the SP-10 gene [9]. Beyond transcription, TDP-43 is also implicated in the maintenance of chromatin structure around long interspersed nuclear elements [17] and DNA damage response [16], both of which are presumably mediated by its DNA-binding capacity. TDP-43, as an RNA-binding protein, associates with more than 6000 target RNAs, including those encoding proteins for neuronal development and function [10, 25–33]. Encompassing a wide range of RNA metabolisms, the RNA-regulatory roles of TDP-43 include RNA splicing, RNA transport, translation [11, 12, 14], and biogenesis of non-coding RNAs [13, 34]. In the following sections, we present an overview of evidence that the diverse functions of TDP-43 are underpinned both by its modular molecular architecture and by its capacity for assembling RNP granules or other protein complexes.

## TDP-43 structure and post-translational modification

**Fig. 2:**
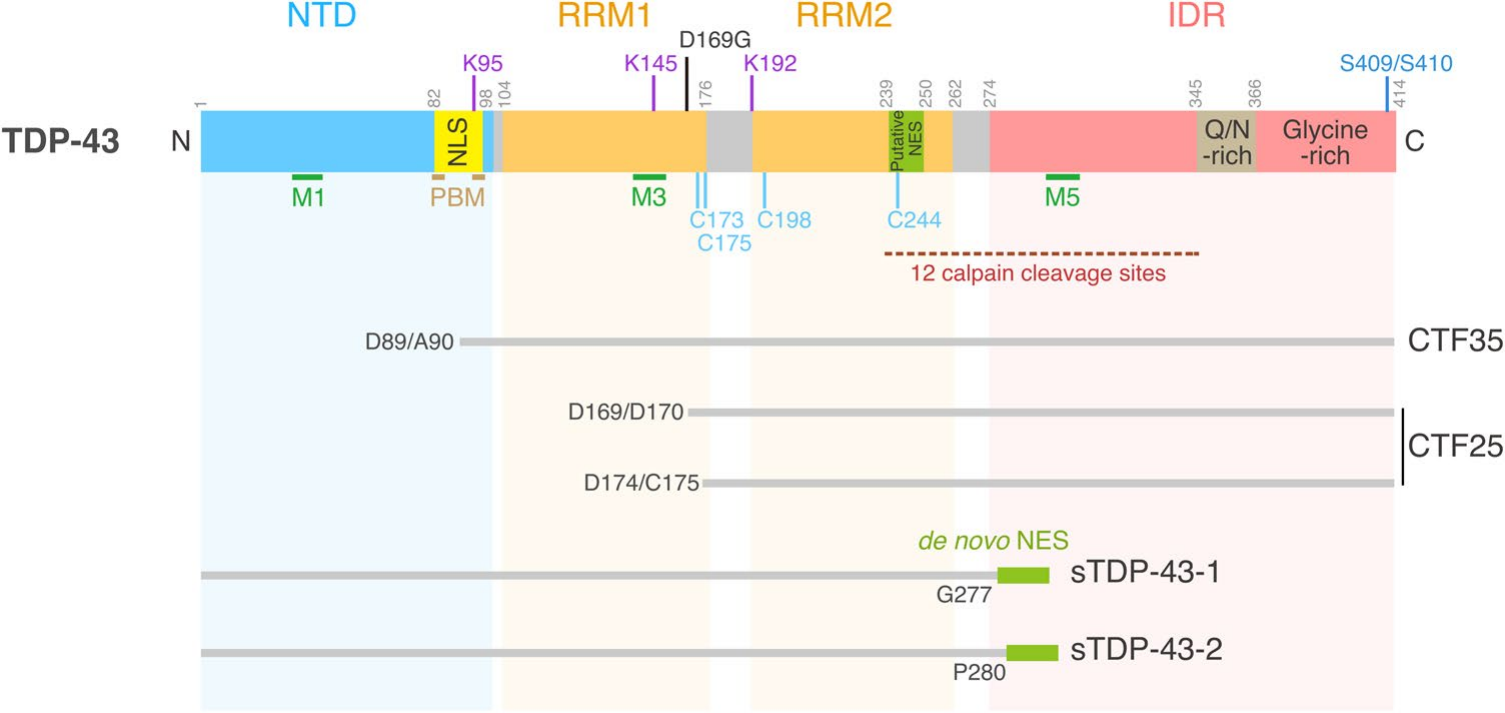
Structure of TDP-43 and its variants. TDP-43 contains 414 amino acid residues and comprises the N-terminal domain (NTD), two RNA recognition motif domains (RRM1 and RRM2), and a C-terminus intrinsically disordered region (IDR). The NTD includes the nuclear localization signal (NLS), overlapping with the poly(ADP-Ribose) (PAR)-binding motifs (PBMs). The NLS is ubiquitinated at K95. The RRM domains are acetylated at K145 and K192, and are subjected to cysteine-mediated disulfide cross-linking at C173, C175, C198 and C244. RRM2 includes a bioinformatically identified putative nuclear export signal (NES). Green bars indicate regions required for mitochondrial localization (M1, M3, and M5). RRM1 contains an ALS-associated mutation, D169G. For more comprehensive views of ALS-associated TDP-43 mutations and ubiquitination sites, see [20] and [42], respectively. The IDR domain contains regions rich in glutamine and asparagine residues (Q/N-rich) and glycine residues (Glycine-rich). The region containing 12 calpain-cleavage sites is indicated by a dashed line. Amino acid numbers are shown above the protein structure. CTF35 and CTF25 are generated by caspase-dependent cleavage. sTDP-43-1 and sTDP-43-2 are the products of neuronal activity-dependent alternative splicing

The multimerization status of TDP-43 governs its physiological and pathological functions. Under normal physiological conditions, the N-terminus of TDP-43 mediates homo-oligomerization, which is necessary for its role in RNA regulation (Fig. 2) [35–38] and can drive liquid–liquid phase separation (LLPS) [39]. The nuclear import receptor importin α recognizes the nuclear localization signal (NLS) embedded in the N-terminus of TDP-43 [40, 40], implying the close coordination of oligomerization and nuclear import. The NLS is subjected to ubiquitination [42], and the ubiquitination at lysine-95 within the NLS likely inhibits nuclear import, targeting TDP-43 for proteolysis in the cytoplasm [43]. The NLS also contains the poly (ADP-Ribose) (PAR)-binding motifs (PBMs) that regulate recruitment to SGs [44]. These observations suggest that the N-terminus domain of TDP-43 mediates the multilayer TDP-43 control of protein multimerization, localization, and stability under normal and stress conditions.

RNA binding of TDP-43 is mediated by the two RNA recognition motif (RRM) domains (RRM1 and RRM2) that reside in the middle of the primary protein structure [11]. RRM1 and RRM2 have differential affinities with different types of RNAs and, under stress conditions, have distinct functions in the assembly and maintenance of nuclear TDP-43 granules [45]. The RRM2 contains a putative nuclear export signal (NES) predicted via bioinformatics [40, 46, 47]. Mutations in the putative NES or inhibition of the nuclear export receptor exportin-1 (XPO1) by leptomycin B treatment lead to nuclear TDP-43 granule formation [40, 45]. However, independent studies have failed to establish TDP-43 as a direct substrate of XPO1 [47–49], and exact mechanisms for the nuclear TDP-43 granule formation caused either by the mutation of the putative NES or leptomycin B treatment remined to be clarified. The RRM domains are also dominant sites of acetylation and cysteine-mediated disulfide cross-linking, two processes that impair the RNA-regulatory functions of TDP-43 [50–52].

The IDR in the C-terminus comprises a glycine-rich domain and a region enriched in glutamine (Q) and asparagine (N). Proteins with IDRs reversibly phase separate into droplets; however, such assembly of IDR proteins may become irreversible when they aggregate due to mutations, prolonged stress, or changes in protein concentration [53]. Supporting this view, most of the ALS-linked TDP-43 mutations are found in this C-terminal IDR [19]. Peptides in this region can efficiently form amyloid-like fibrils in vitro that can exhibit prion-like infectious seeding ability in cells expressing the soluble TDP-43 [54–57]. Under pathological conditions, TDP-43 is hyperphosphorylated, ubiquitinated, and cleaved to generate aggregation-prone C-terminal fragments (CTFs) (Fig. 2) [58–61]. Intracellular aggregation of the full-length TDP-43 that is recognized by the antibody against S409/S410 phosphorylation (p409/410) precedes the generation of TDP-43 CTFs, suggesting that CTFs are not essential for the formation of intracellular TDP-43 aggregates [62].

## TDP-43 granules in the nucleus and cytoplasm

*Nuclear bodies (NBs)*

In mammalian cells, both endogenous TDP-43 and exogenously expressed TDP-43 at a normal endogenous level show a demixed distribution as rounded particles in the nucleus [7, 63]. TDP-43 is a known component of several kinds of membraneless nuclear structures (i.e. nuclear bodies, or NBs), which are enriched with specific nuclear factors in continuous exchange with the surrounding nucleoplasm [64], such as paraspeckles (PSs) [65] and Cajal bodies [66–68]. When incorporated into the PSs, TDP-43 is prevented from regulating alternative polyadenylation of pluripotency factor mRNAs in embryonic stem cells, thereby influencing cellular differentiation [69]. On the inactive X chromosome, a set of RNA-binding proteins including TDP-43 form heteromeric condensates with the long non-coding RNA Xist to initiate and maintain gene silencing [70]. These observations elucidate the close linking of the intranuclear phase transition of TDP-43 to its RNA-regulatory roles. The granular appearance of nuclear TDP-43 is enhanced when cells are under stress, assembling dynamic and reversible TDP-43-containing NBs [45, 51, 71]. Upon arsenite treatment, TDP-43 associates with distinct RNA species, such as long non-coding RNA NEAT1 or short tRNAs, for NB assembly via its two RRM domains [45]. Sequestration of TDP-43 into stress-induced NBs may be a neuroprotective strategy, because the recruitment of TDP-43 to NBs is compromised by the ALS-causing D169G mutation in RRM1, resulting in the incorporation of the mutant TDP-43 into SGs in the cytoplasm [45]. TDP-43 is colocalized with PSs in the spinal motor neurons of sporadic ALS patients [67], and excessive PS formation has been observed in ALS-FUS [72]. Although PS hyper-assembly has been shown to have a protective effect [34], the significance of TDP-43 recruitment to PSs in ALS pathology remains elusive. An intranuclear spherical shell structure that is formed by an RNA-binding-deficient TDP-43 and includes HSP70 chaperones in the core was recently identified and termed anisosome [73]. This HSP70 chaperone-dependent droplet harbors a liquid crystalline property and prevents RNA-free TDP-43 from forming round cytoplasmic droplets and converting into gel/solid-states, which might be precursors of the TDP-43 aggregates observed in neurodegenerative diseases, including ALS. Besides its RNA-regulatory roles, TDP-43 is also implicated in DNA damage repair. TDP-43 depletion causes an accumulation of DNA double-strand breaks (DSBs), while TDP-43 overexpression is protective against DSBs [16, 74–77]. TDP-43 is rapidly recruited at DSB sites upon induction of DNA damage to stably interact with factors regulating DNA damage response and non-homologous end joining [74, 75]. The association between the role of TDP-43 in DNA damage repair and TDP-43-containing NB assembly remains to be determined.

*RNP transport granules*

Membraneless organelles formed via condensation of protein–RNA complexes, or phase transition, is relevant for the long-range transport of biomolecules particularly for large-sized and polarized cell types, including motor neurons, where transcription in the nucleus and translation at the synapse can be as far apart as the entire length of the spinal cord, arms, or legs. A fraction of cytoplasmic TDP-43 in neuronal cells functions as a component of RNP granules that undergo microtubule-dependent transport along the axon [12, 78]. Exogenous expression of human TDP-43 in the fly motor neurons and rodent primary cortical neurons results in the formation of RNP granules containing human TDP-43 in the axons [12, 78]. The TDP-43-containing RNP granules are transported bidirectionally, with brief pauses, for long distances, and display liquid-like properties such as fusion, fission, and exchange of TDP-43 with the cytoplasmic soluble TDP-43 pool. The biophysical traits of these TDP-43 granules vary depending on the axonal location; the TDP-43 granules in the mid-axon display a more enhanced motility, a rapid molecular exchange rate and higher sphericity, while those in the proximal axon tend to have limited motility, a lower molecular exchange rate, and more irregular contours [78]. LLPS mediates TDP-43 granule formation in the mid-axon; treatment with 1,6-hexanediol, which disrupts weak hydrophobic interactions in RNP granules [79], rapidly and reversibly dissolves TDP-43 granules in the mid-axon without affecting the integrity of those in the proximal axons. Remarkably, the ALS-linked mutations in the IDR increase the viscosity of the granules and promote retrograde, but not anterograde, transport, resulting in the accumulation of TDP-43-containg granules in the proximal axons [12, 78]. TDP-43 also displays granular localization in the dendritic arbors, enhanced by neuronal depolarization [14]. ALS-linked TDP-43 mutations reduced the depolarization-dependent dendritic localization [80]. Overall, the liquid-like properties of TDP-43-containing RNP transport granules are a critical determinant of the distance that mRNAs can travel along the axons and dendrites, and loss of these properties may underlie ALS pathology by affecting local proteomes in axons and dendrites of motor neurons.

*SGs*

To sustain cell survival, global repression of translation occurs in response to cellular stresses. SGs are cytoprotective membraneless organelles comprising RNA–protein complexes, and seen in the cytoplasm of cells under stress [81, 82]. TDP-43 is not a ubiquitous component of SGs, but is recruited in response to many, but not all, stressors [51, 83–85]. The recruitment of TDP-43 to SGs is promoted by the binding of PAR, a negatively charged biopolymer, to the PAR-binding motif (PBM) embedded in the NLS of TDP-43 (Fig. 2) [44]. The incorporation of TDP-43 into SGs protects it against pathological phosphorylation of the IDR at S409/S410, as shown by the formation of cytoplasmic granules distinct from SGs under stress by TDP-43 mutants defective in binding to PAR, and more prone to show the pathological phosphorylation [44]. Upon stress, the amount of TDP-43 recruited to SGs is also influenced by the assembly of TDP-43-containing NBs in the nucleus. The stress-dependent recruitment of TDP-43 to NBs is diminished by the ALS-causing D169G mutation within RRM1, while the formation of TDP-43-containing SGs is conversely significantly enhanced in the cytoplasm, raising the possibility that assembly of TDP-43-containing NBs works as the first line of defense against stress to prevent excessive recruitment and accumulation of TDP-43 in cytoplasmic SGs [45]. Although recruitment of TDP-43 to SGs may in the short term be beneficial, exposure to high levels of stress for a prolonged period leaves aggregates of pathologically phosphorylated TDP-43 after SG resolution [44]. Moreover, chronic optogenetic induction of SG assembly leads eventually to the deposition of pathologically phosphorylated TDP-43-containing aggregates and causes cytotoxicity [85]. These observations suggest that TDP-43 having experienced a prolonged SG incorporation may become a precursor for pathological TDP-43 aggregates. On the other hand, recent reports have described pathological TDP-43 granules devoid of SG-resident proteins and associated with cytotoxicity [6, 7, 44]. The extent of the contribution by SGs to the formation of pathological TDP-43 aggregates in ALS and a subtype of frontotemporal lobar degeneration (FTLD-TDP) remains to be evaluated.

*TDP-43 in myo-granules*

Cytoplasmic TDP-43 granules have been shown to play important physiological roles in skeletal muscles. Reduced levels of TDP-43 in skeletal muscles lead to age-related muscle weakness in mice and flies [86–88] and to muscle degeneration in zebrafish [89]. While TDP-43 is abundant in the nuclei of C2C12 myoblasts and primary mouse myoblasts, during their differentiation into multinucleated myotubes, cytosolic TDP-43 increases, resulting in the formation of 50–250 nm assemblies with amyloid-like properties, called myo-granules [90]. TDP-43 in myo-granules, which bind to mature mRNAs encoding sarcomeric components, is essential for skeletal muscle cell differentiation in culture and skeletal muscle regeneration in mice [90]. Analogously, in axotomized motor neurons in mice, cytoplasmic TDP-43 granules transiently accumulate that colocalize strongly with the RNA transport granule marker Sauften and moderately with the generic SG marker TIA-1 [91], implying that cytoplasmic TDP-43 redistribution is a part of the normal and physiological response to cellular injury. These findings have demonstrated that the multimerization status of TDP-43 differs depending on physiological cellular conditions and that TDP-43 oligomers can be both beneficial and harmful, depending on the cell-type and possibly the age. The ability of TDP-43 in myo-granules to seed TDP-43 aggregation in motor neurons, via its prion-like ability to spread across neuronal connectivity [92], remains undetermined.

*Pathological TDP-43 aggregates*

Cytoplasmic inclusions of TDP-43, appearing as rounded or skein-like inclusions in degenerating neurons, are a reliable pathological hallmark of ALS and FTLD-TDP [93, 94]. In FTLD-TDP, TDP-43 pathology can be categorized into four subtypes (types A–D) based on the histology of the TDP-43-positive structures, and disease severity is correlated with the distinct forms of pathological TDP-43 [95, 96]. The distinct histological traits of TDP-43 aggregate suggest multiple pathways for aggregation. Indeed, in cultured cells, cytoplasmic TDP-43 aggregation is driven by at least two distinct pathways upon expression of inherited ALS/FTLD causative genes: RNA-binding protein-mediated LLPS promoting granular-type aggregation and histone deacelylase 6 (HDAC6)-mediated aggresome formation promoting skein-like aggregation [97]. In the spinal cord of patients with ALS, most of the phosphorylated TDP-43 inclusions show significant skein-like immunoreactivity of lysine-145 acetylation in RRM1, which may be promoted by oxidative stress [50]. This implies that the cellular stresses specify the form of TDP-43 aggregates. The dipeptide repeat proteins expressed from the *C9orf72* locus carrying repeat expansions, a cause of familial ALS, also causes TDP-43 aggregation [43, 98–102]. Intracellular TDP-43 is aggregated in a self-templating manner when the cultured cells are treated with seeds isolated from the brains of patients with ALS and FTLD-TDP, as well as synthetic peptide-derived TDP-43 CTF fibrils; this suggests that the alternate pathological TDP-43 conformations in ALS and FTLD-TDP could also arise from the prion-like properties of TDP-43 [57, 62, 103, 104]. Of note, in a synthetic peptide-dependent TDP-43 aggregation assay, phospho-deficient mutations in the CTF have little effect on the aggregation propensity of TDP-43 [57], hinting at the possibility of a toxic TDP-43 variant undetectable via conventional phospho-CTF immunostaining. A recent report of neurotoxicity observed in the presence of cytoplasmic TDP-43 granules lacking S409/S410 phosphorylation supports this view [6]. Intercellular transfer of TDP-43 has been demonstrated in vitro [103–106]. In vivo transmission of TDP-43 along neuronal connectivity was first demonstrated using animal models by injecting human brain-derived FTLD-TDP extract into mouse brain overexpressing TDP-43 [92]. Microvesicle/exosome-dependent intercellular TDP-43 transport might mediate transmission and de novo formation of pathological TDP-43 aggregates in a distant brain area [62, 103], which may not be dependent on SG formation because the assembled phosphorylated TDP-43-positive inclusions did not colocalize with SG markers in immunofluorescence [92].

## TDP-43 and selective neuronal vulnerability

In sporadic ALS, pathological TDP-43 phosphorylation recognized by the pS409/S410 antibody is observed throughout many areas of the central nervous system (CNS) [107], showing that ALS is a multisystem TDP-43 proteinopathy. Studies using animal models also suggest that the deposition of cytoplasmic aggregates does not always accompany TDP-43 neurotoxicity [24, 108]. These observations question the extent to which cytoplasmic TDP-43 aggregates, as end products, explain the selective vulnerability of motor neurons in ALS. Although the answer is largely elusive at present, several studies have begun to reveal neuron-specific properties and regulatory function of TDP-43, including the alternative splicing of TDP-43 mRNA and proteolytic cleavage of TDP-43 protein. In both physiology and pathology, motor neuron-specific properties of TDP-43 could modify functions of TDP-43-containing membraneless organelles, and would be key to explaining selective vulnerability of motor neurons in ALS.

*Neural activity-dependent alternative splicing of TDP-43*

Motor neurons are large cells with large membrane surface areas, which demand high levels of energy for generating an action potential, as well as for maintaining homeostatic ionic gradients across the plasma membrane in the resting state. The energetic demand is particularly high in large fast-fatigable motor neurons, the most vulnerable neuronal type in ALS [109], and these neurons are prone to hyperexcitation due to low GABAA and glycine receptor expression [110]. Accordingly, cortical hyperexcitability and mislocalization of TDP-43 are salient and highly conserved features of ALS. Recently, hyperexcitability was found to lead to the expression of two shortened splice isoforms of TDP-43 (sTDP-43-1 and sTDP-43-2), wherein the entire glycine-rich domain of TDP-43 is replaced by short tails generated by the inclusion of a new exon encoding a unique 18-amino acid C-terminus not found in the wild-type full-length TDP-43 (Fig. 2) [111]. The sTDP-43-1 isoform is prone to cytoplasmic localization due to a de novo NES created by the neural activity-dependent splicing. In rodent primary mixed cortical neurons, overexpression of sTDP-43-1 is neurotoxic, and promotes the cytoplasmic deposition and nuclear clearance of endogenous TDP-43 through N-terminus- and/or RRM-mediated aggregation. Moreover, significant expression of the sTDP-43-1 isoform is detectable in several different regions of the human CNS, including spinal motor neurons. Major challenges in proving a causal link between neuronal hyperexcitability and cytoplasmic TDP-43 aggregation in the selective vulnerability of human motor neurons in ALS include the elucidation of mechanisms underlying the neural activity-dependent alternative splicing and effects of the sTDP-43 isoforms on TDP-43-containing membraneless organelles.

*Ca* ^*2*+^ *-dependent cleavage of TDP-43*

**Fig. 3:**
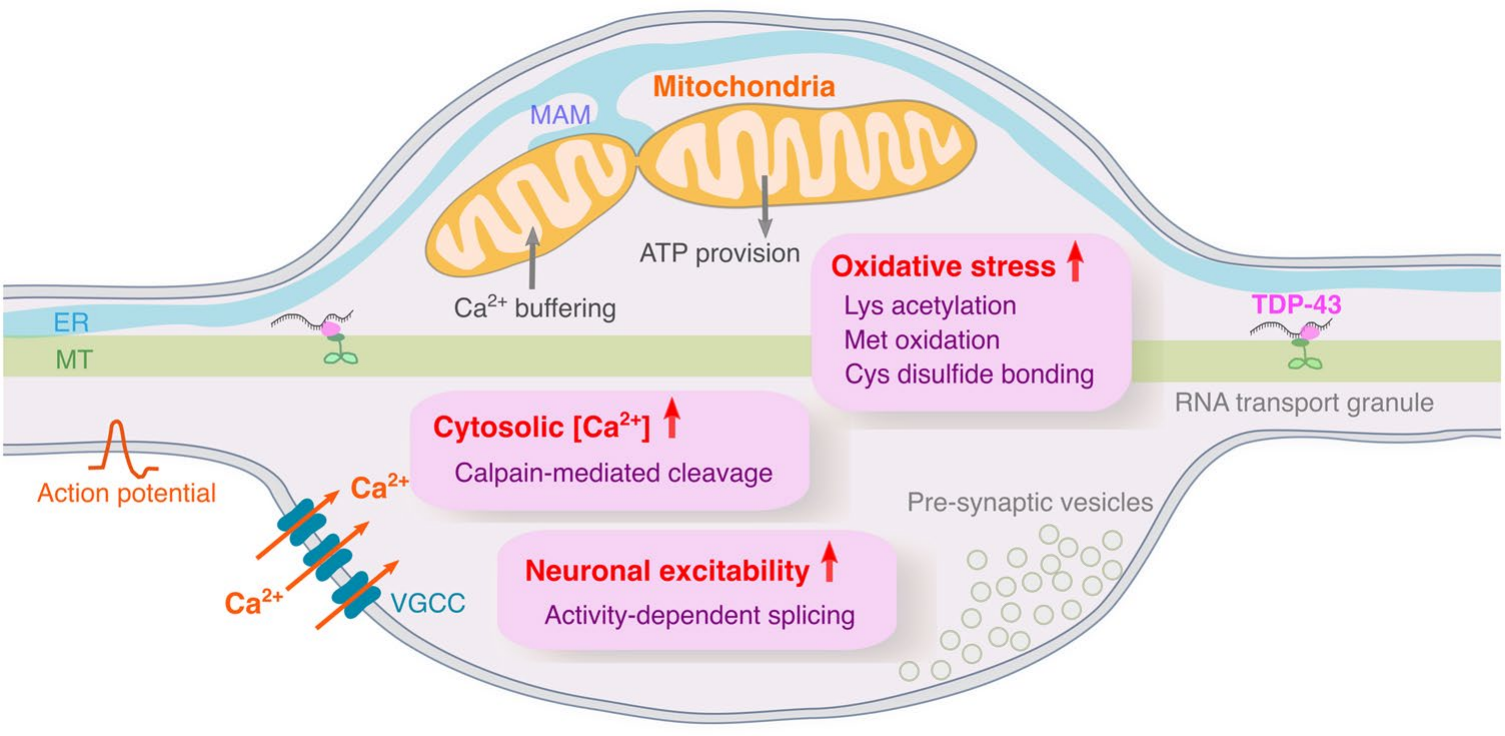
Possible upstream mechanisms that promote pathological TDP-43 phase transition and aggregation. Cellular redox activity influences self-interaction, aggregation, and cytotoxicity of TDP-43 by promoting lysine acetylation, methionine oxidation, and cysteine disulfide bonding of TDP-43. An exaggerated Ca^2+^ influx elevates cytosolic Ca^2+^ concentration, which leads to calpain-mediated cleavage of TDP-43 that, in turn, generates aggregation-prone TDP-43 fragments. These post-translational TDP-43 modifications may frequently occur at mitochondria-rich subcellular compartments, including, but not restricted to, pre-synaptic axon swellings (illustrated), where ROS is actively produced due to high energy metabolism and efficient Ca^2+^ buffering is needed. Neuronal hyperexcitability drives the expression of two shortened splice isoforms of TDP-43 (sTDP-43-1 and sTDP-43-1), which are also aggregation-prone. The mechanisms underpinning how neuronal hyperexcitability drives TDP-43 alternative splicing are elusive. *ER* endoplasmic reticulum, *MT* microtubule, *VGCC* voltage-gated calcium channel

Elevated intracellular Ca^2+^ levels caused by excessive stimulation of glutamate receptors have been implicated in the selective vulnerability of neurons in ALS [112–115]. Glutamate-mediated excitotoxicity is associated with the stoichiometry of both Ca^2+^-permeable subunits GluA1, 3, and 4 and the typically Ca^2+^-impermeable subunit GluA2 [116–124]. TDP-43 pathology in sporadic ALS is correlated with the downregulation of an RNA-editing enzyme, adenosine deaminase acting on RNA 2 (ADAR2), which edits GluA2 pre-mRNA to produce the Ca^2+^-impermeable GluA2 subunit [125–128], suggesting that exaggerated Ca^2+^ influx could lead to TDP-43 aggregation. The observation that TDP-43 is subject to proteolysis in a Ca^2+^ -dependent manner provides a mechanistic link between the exaggerated Ca^2+^ influx and TDP-43 aggregation [129] (Fig. 3). In mouse motor neurons, TDP-43 is cleaved by calpains (Ca^2+^-dependent cysteine proteases) at the C-terminus, which is distinct from the caspase-dependent cleavage sites (Fig. 2) [61, 130], to generate an aggregation-prone proteolytic product [129]. Calpain-dependent TDP-43 fragments are detectable in the spinal cord and brain of patients with ALS. Thus, the calpain-mediated TDP-43 cleavage is a crucial downstream target of an excessive intracellular Ca^2+^ load, potentially affecting TDP-43-containing membraineless organelles via generation of aggregation-prone TDP-43 fragments and contributing to the selective vulnerability of motor neurons in ALS.

*TDP-43 and mitochondria*

The high energetic demand of motor neurons is met by ATP provision via mitochondrial metabolism. Inevitably, mitochondria are major sources of reactive oxygen species (ROS) within most mammalian cells, and modulation of cellular redox activity has been shown to influence self-interaction, aggregation, and cytotoxicity of TDP-43 [131–133]. Oxidative stress-induced cysteine oxidation and disulfide bond formation in the RRM domains lead to impaired splicing function and reduced solubility of TDP-43 [51, 52]. The RRM1 is also acetylated at lysine-145 in response to oxidative stress by histone acetylase cAMP response element-binding (CREB)-binding protein (CBP), impairing RNA-binding and promoting deposition of skein-like TDP-43 inclusions [50]. Furthermore, methionine oxidization in the IDR of TDP-43 affects its ability to self-assemble into an oligomeric structure [134]. These observations suggest that high mitochondrial metabolism could alter the phase behavior of TDP-43 via ROS production especially in a mitochondrial-rich subcellular compartment, such as pre-synaptic terminals [135, 135] (Fig. 3). Another essential function of mitochondria is the maintenance of cellular Ca^2+^ homeostasis, which is regulated through interactions between the endoplasmic reticulum (ER) and mitochondria via a region in the ER called mitochondria-associated membranes (MAM) [137, 138]. MAM is implicated in neurodegenerative diseases [139–145]. Tightening of MAM-mediated ER-mitochondria contacts can cause Ca^2+^ overload in mitochondria, whereas its loosening diminishes mitochondrial ATP synthesis and increases cytosolic Ca^2+^ [146–149]. Increases in cytosolic Ca^2+^ could in turn induce calpain-mediated cleavage of TDP-43, leading to TDP-43 aggregation [129] (Fig. 3); a direct link between MAM disruption and calpain-dependent TDP-43 cleavage, however, remains to be demonstrated. Taken together, mitochondria are key organelles influencing phase behaviors of TDP-43 through ROS generation and maintenance of Ca^2+^ homeostasis, whose dysfunction could contribute to selective vulnerability of motor neurons in ALS.

Of note, induced pluripotent stem cell (iPSC)-derived motor neurons from patients carrying TDP-43 mutation display high glutamate-induced Ca^2+^ release and delayed buffering of cytosolic Ca^2+^ [150], suggesting that TDP-43 regulates mitochondria, as well as being influenced by them. Indeed, endogenous TDP-43 is detectable in the mitochondria of brain samples with or without FTLD-TDP pathology, using immuno-electron microscopy [151]. Furthermore, in brain samples from patients with FTLD-TDP and ALS-FTLD-TDP, electron dense TDP-43-positive protein aggregates and impaired mitochondrial morphology have been observed [151]. These human studies suggest the mitochondrial roles of TDP-43, but in the studies using cellular and animal models, conflicting results have been reported for direct involvement of TDP-43 in mitochondrial respiratory complex and ATP synthesis [145, 151–155]. Further studies are thus necessary to understand the mitochondrial function of TDP-43 expressed at physiological levels. TDP-43-mediated mitochondrial control has also been suggested from overexpression experiments. Overexpression of TDP-43 disrupts MAM and increases cytosolic Ca^2+^ at the expense of mitochondrial Ca^2+^ storage [142]. The TDP-43-dependent MAM disruption is mediated via the activation of GSK -3β, although the mechanism whereby excessive TDP-43 is sensed by GSK-3β remains unknown [142]. In cellular and mice models, overexpression of TDP-43 also leads to invasion of TDP-43 into the mitochondria, the release of mitochondrial DNA (mtDNA) into the cytoplasm, and inflammation driven by the cytoplasmic DNA sensor cyclic guanosine monophosphate (GMP)-AMP synthase (cGAS)/STING pathway [156]. The mitochondrial dysfunctions caused by TDP-43 overexpression may be relevant to an understanding of TDP-43 pathology in ALS, and whether the TDP-43-dependent release of mtDNA and Ca^2+^ from mitochondria involves TDP-43 phase transition remains an open question.

*Stability and dynamics of TDP-43 in motor neurons*

Mainly due to anatomical inaccessibility, TDP-43 dynamics have rarely been explored in vivo in mammalian motor neurons. Direct observation of TDP-43 in live motor neurons is, however, feasible in an optical-friendly vertebrate model, zebrafish [23, 24]. We have found that optogenetic oligomerization of TDP-43 with the C-terminally tagged CRY2olig [157] resulted in an efficient increase in cytoplasmic TDP-43 in the spinal motor neurons of zebrafish, leading eventually to the accumulation of cytoplasmic TDP-43 aggregates positive for pS409/S410 immunoreactivity [24]. Intriguingly, in epithelial cells or differentiated myofibers, this light-dependent cytoplasmic shift of TDP-43 is not as efficient or is almost absent. While the mechanism underlying this motor neuron-specific cytoplasmic TDP-43 accumulation remains to be determined, this observation implies that motor neurons possess a unique mechanism for responding to oligomeric TDP-43. One possible explanation for the efficient cytoplasmic TDP-43 accumulation in motor neurons is that oligomeric TDP-43 is more stable in the cytoplasm of motor neurons than in that of other cell types. This idea might be consistent with the observations that TDP-43 has a longer half-life in primary rodent cortical neurons (approximately 18 h) [158], than in fibroblasts, HeLa cell lines (4–12 h) [159], and Neuro2a cell lines (12.6 h) [160]. It is also worth noting that in the cytoplasm, the TDP-43-containing transport granules display differential TDP-43 exchange rates between the mid and proximal regions of axons [78], suggesting that the dynamics and stability of TDP-43 granules differ locally, within the axons and possibly dendrites. The stability and dynamics of TDP-43 in motor neurons thus requires further study. How motor neurons control global and local levels of TDP-43 is a pivotal question to be addressed in the future, and is likely relevant to understanding the selective vulnerability of motor neurons in ALS.

## Outlook

Here, we enumerated different forms of TDP-43 granules in different intracellular locations and contexts. However, these various forms of TDP-43 are insufficient in providing a complete understanding of multifaceted TDP-43 dynamics, in healthy and diseased states. Therefore, it is necessary to explore further TDP-43 granules and their regulatory mechanisms that have not yet been discovered. Aberrant phase behavior of TDP-43 in RNP granules results in at least three reasonably conceivable consequences, differing in their gain-/loss-of-function nature: (1) generation of toxic TDP-43 oligomers or aggregates that further propagate via phase transition in a dominant fashion, (2) loss/reduction of proteins that are otherwise normally expressed, due to TDP-43-mediated regulation, and (3) generation of truncated translation products of abnormally spliced transcripts causing proteostatic cellular stress. These multiple phenotypes are likely to occur simultaneously rather than sequentially. Thus, an understanding of the upstream cellular events causing abnormal TDP-43 phase transition may be as important as the conventional gain-of-function versus loss-of-function dichotomy. An emerging figure, while largely omitted from this review, is the multisystem nature of ALS and FTLD, where TDP-43 pathology in degenerating neurons has been linked to extracellular factors, including inflammation, microglial toxicity, and intercellular *C9orf72*-derived dipeptide transmission [43, 161–163]. Studies investigating the precise mechanisms of upstream events of TDP-43 pathology at the multisystem levels therefore warrant intensive efforts and potentially provide effective treatment targets applicable to a wide range of different ALS subtypes and other TDP-43 proteinopathies.
